# Case report of a triple vector-borne infection in a dog: co-infection with *Anaplasma phagocytophilum*, *Babesia* spp., and *Dirofilaria repens* in North-Eastern Poland

**DOI:** 10.1186/s12917-025-04889-4

**Published:** 2025-07-05

**Authors:** Ismena Gałęcka, Aleksandra Platt-Samoraj

**Affiliations:** Department of Epizootiology, Faculty of Veterinary Medicine, University of Warmia and Mazury in Olsztyn, Oczapowskiego 13, 10-719 Olsztyn, Poland

**Keywords:** Blood smear, Tick borne disease, Co-infection, Canine infectious diseases

## Abstract

**Background:**

This is the first case of triple co-infection with vector-borne diseases confirmed by blood smear results and molecular confirmation of the anaplasmosis and dirofilariosis in a dog from North-Eastern Poland.

**Case presentation:**

A 4-year-old, uncastrated male Central Asian Shepherd Dog with symptoms of apathy and lack of appetite was diagnosed with triple vector disease infection. Microscopic examination of a blood smear revealed the presence of *Babesia* spp., *Anaplasma phagocytophilum* and microfilariae. PCR confirmed the presence of *A. phagocytophilum* and *Dirofilaria repens*. Clinical examination revealed elevated core body temperature and thrombocytopenia. Treatment for the identified pathogens was initiated. Complete improvement of clinical condition was observed on the second day of treatment.

**Conclusion:**

The detection of three different pathogens in a blood smear is not often reported, but it can significantly speed up the diagnosis and initiation of targeted treatment. It should be confirmed using molecular methods, which are commonly used in companion animals, especially for diagnosing co-infections.

## Background

The diagnosis and treatment of vector-borne diseases pose an increasing challenge for veterinarians. Climate change and the geopolitical situation in Europe contribute to the emergence of pathogens in previously unaffected regions [1]. The impact of climate change may be confirmed by the diagnosis of tick-borne diseases in dogs during the winter months (November-February) from North-Eastern Poland [2]. In some cases, these pathogens were historically absent, while in others, only sporadic occurrences have been reported.

The etiological factor of subcutaneous dirofilariosis is *Dirofilaria repens* [3, 4], a nematode transmitted by mosquitoes [4]. Clinically, it often presents as subcutaneous nodules [4] or may be diagnosed incidentally during blood smear, cytology or histopathology tests [3]. The growing number of reported cases might indicate that dirofilariosis is currently the most rapidly spreading zoonosis in Europe [4].

Babesiosis is a multi-organ disease caused by parasites of the order Piroplasmida, typically *Babesia* spp. [5]. The disease poses a significant health threat to both humans and animals, with incidence rates rising annually [5]. Babesiosis may occur with subclinical infections or manifest itself as a multi-organ disease with potentially lethal consequences [5, 6]. In Europe, canine babesiosis is caused mainly by *Babesia canis* and *Babesia vogeli*, although the prevalence of infections caused by *Babesia gibsoni* is on the rise [6].

Granulocytic anaplasmosis is diagnosed mainly in northern and central Europe [7]. The etiological agent is *Anaplasma phagocytophilum*, and *Ixodes ricinus* is considered as the main vector [7]. The prevalence of anaplasmosis in Europe varies widely from 3 to 57% [7]. These variations can be largely attributed to differences in diagnostic methodologies, particularly the use of serological assays, which may detect prior exposure to the pathogen without confirming an active infection or a clinical disease [7].

Up to date, there is no official or indexed report available confirming a case of triple co-infection with *Babesia* spp., *D. repens*, and *A. phagocythilum* in a blood smear of a dog from the Warmian-Masurian Voivodeship, Poland, with no history of travel outside its home region. *A. phagocytophilum* and *D. repens* infection was additionally confirmed by PCR method performed in the reference laboratory.

## Case presentation

On 14 October 2022, a 4-year-old, uncastrated male Central Asian Shepherd Dog with a body weight of 53 kg was admitted to the Veterinary Polyclinic at the Faculty of Veterinary Medicine of the University of Warmia and Mazury in Olsztyn, Poland.

The dog resided in a rural area in the proximity of a forest near Olsztyn in the Warmian-Masurian Voivodeship, Poland, and had never traveled outside the home region. It was regularly vaccinated against infectious diseases and rabies in accordance with the applicable WSAVA guidelines. Regular tick prophylaxis involved the use of collars with imidacloprid and flumethrin, combined with spot-on applications of fipronil.

The clinical examination revealed apathy, a body temperature of 39.8 °C, light pink mucous membranes and capillary refill time > 2 s. Venous blood was collected from the cephalic vein into tubes containing EDTA and a clot activator. Morphological and biochemical analyses were conducted using the IDEXX Catalyst One Veterinary Chemistry Analyzer and the IDEXX ProCyte Dx Hematology Analyzer (IDEXX Laboratories, Inc., Maine, United States). Blood smears were stained using the Hemavet kit (Kolchem, Łódź, Poland). The SNAP 4Dx Plus test for *Dirofilaria immitis* antigen, antibodies against *A*. *phagocytophilum*/*platys*, *Borrelia burgdorferi*, and *Ehrlichia canis/ewingii* (IDEXX Laboratories, Inc., Maine, United States) was also performed. The morphological examination revealed lymphopenia (0.56 K/µL), monocytosis (1.14 K/µL), significant thrombocytopenia (39 K/µL), increased mean thrombocyte volume (16.8 fL), decreased thrombocrit (0.07%), and decreased reticulocyte hemoglobin concentration (18.6 pg). Biochemical tests revealed only a mild increase in alanine aminotransferase (ALT) concentration (55 U/L; reference range: ≤50 U/L) and hemolysis. Detailed results are presented in Table 1.

**Table 1:** The results of hematological and biochemical tests

Parameter			Date	
Parameter	Unit	Reference range	14/10/2022	13/11/2022
RBC	M/µL	5.65–8.87	8.27	6.78
HCT	%	37.3–61.7	49.1	43.9
HGB	g/dL	13.1–20.5	18.6	15.1
MCV	fl.	61.6–73.5	59.4	64.7
MCH	pg	21.2–25.9	22.5	22.3
MCHC	g/dL	32-37.9	37.9	34.4
RDW	K/ µL	13.6–21.7	19.3	18
RETIC	K/ µL	10–110	24	80
WBC	K/ µL	5.05–16.76	9.58	12.61
NEU	K/ µL	2.95–11.64	7.84	8.28
LYM	K/ µL	1.05–5.1	**0.56**	3.32
MONO	K/ µL	0.16–1.12	**1.14**	0.51
EOS	K/ µL	0.06–1.23	**0**	0.44
BASO	K/ µL	0-0.1	0.04	0.06
PLT	K/ µL	148–484	**39**	317
MPV	fL	8.7–13.2	**16.8**	11.1
PCT	%	0.14–0.46	**0.07**	0.35
RETIC-HGB	pg	22.3–29.6	**18.6**	**21.8**
ALKP	U/L	23–212	66	50
ALT	U/L	10–125	52	23
AST	U/L	0–50	**55**	27
BUN	mg/dL	7–27	14	7
CREA	mg/dL	0.5–1.8	1.2	0.8

Parameters outside the reference values are marked in bold.

**Fig. 1:**
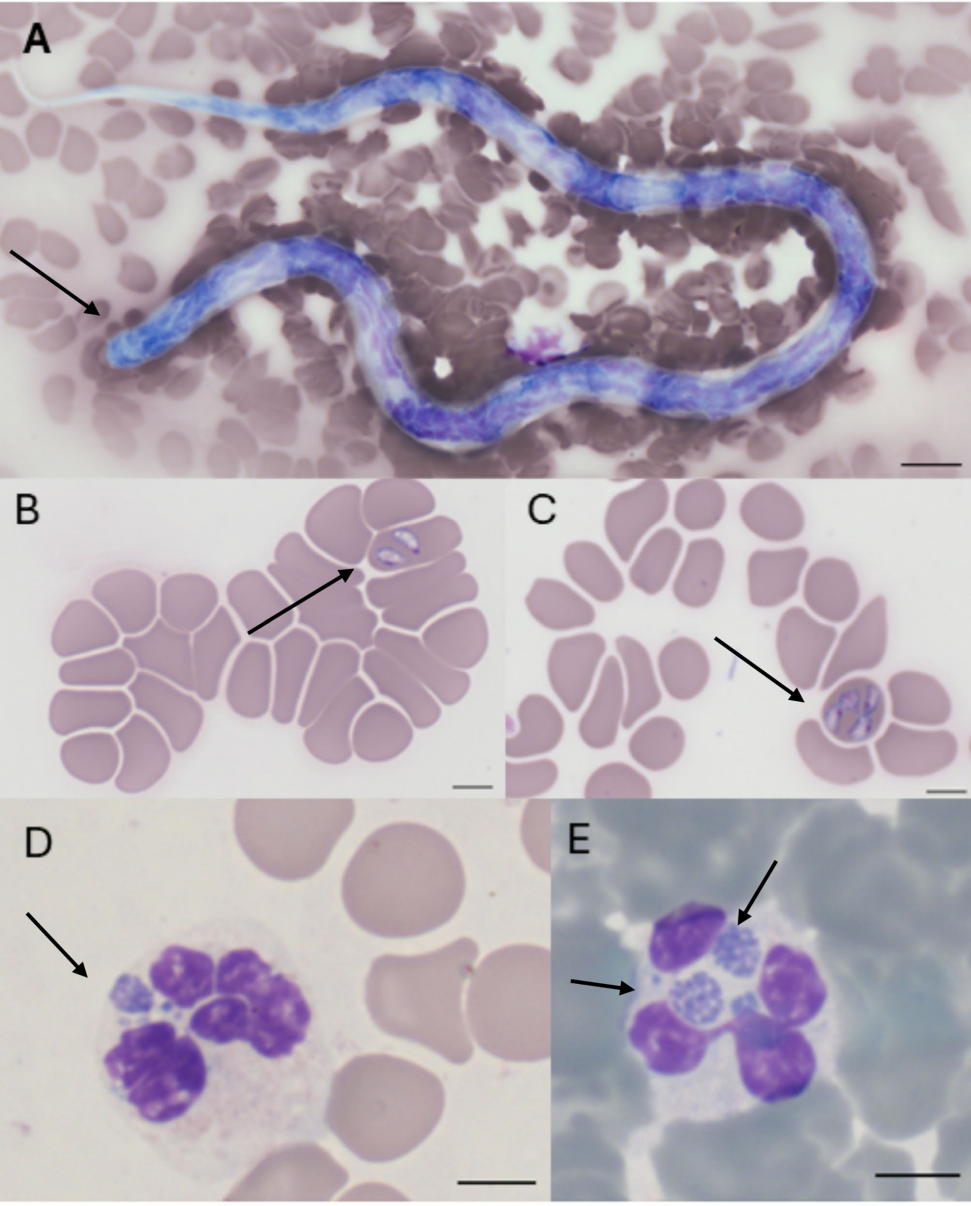
Blood smear. **A**) microfilaria; **B** and **C**) *Babesia* spp.; **D** and **E**) *A. phagocytophilum*. Scale bar: 5 μm. Zeiss Axio Imager Z1 microscope (Zeiss, Germany)

The SNAP 4Dx Plus test yielded a positive result for *A. phagocytophilum/platys*. After approximately 30 min, a weakly stained dot suggested the presence of a *D. immitis* antigen. The blood smear collected into an EDTA tube revealed the presence of large-sized *Babesia* spp. in erythrocytes (pear-shaped inclusions; Fig. 1B, C), morulae with *A. phagocytophilum* in neutrophils (basophilic intracytoplasmic inclusions; Fig. 1D, E), and microfilariae between blood cell components (obtuse-rounded cephalic margin and long sharp tail [8], Fig. 1A). To confirm these findings, the blood sample was transported to IDEXX Laboratories Sp. z o.o. (Division of IDEXX Laboratories, Warsaw, Poland) for PCR testing. The sample tested positive for *A. phagocytophilum* and *D. repens*. The owners decided not to confirm the *Babesia* spp. by PCR for financial reasons.

Metamizole (25 mg/kg, intravenous, Pyralgivet, VET-AGRO P.W. Sp. z o. o., Lublin, Poland), dexamethasone (0.5 mg/kg, intravenous; Rapidexon, Eurovet Animal Health B.V., Bladel, the Netherlands), imidocarb dipropionate (6 mg/kg, subcutaneous; Lovacarb, Lovapharm Consulting B.V., Eindhoven, the Netherlands), and a multi-electrolyte solution (intravenous) were administered during the first visit. The follow-up treatment involved doxycycline (5 mg/kg, twice daily, orally, for 4 weeks; Doxybactin, Le Vet Beheer B.V., Oudewater, Netherlands) and a probiotic (3 capsules, once daily; Flora Defense, REGIS sp. z o.o., Krakow, Poland). The dog exhibited significant clinical improvement the following day, with balance problems resolved and a near-complete return to normal appetite and behavior. Complete improvement of clinical condition was observed on the second day of treatment.

The treatment against *D. repens* was initiated one week after the first visit when the parasite was confirmed by PCR. The treatment protocol consisted of monthly administration of a spot-on imidacloprid preparation at a minimum dose of 10 mg/kg and moxidectin at 2.5 mg/kg (Advocate, Bayer, Leverkusen, Germany) for six months. Given the owners’ report of past allergic reactions (to penicillin), prednisolone was introduced prophylactically in decreasing weekly doses (0.5 mg/kg twice daily in the first week, 0.5 mg/kg once daily in the second week, 0.5 mg/kg once daily every 2–3 days in the third and fourth weeks; Prednicortone, Le Vet Beheer B.V., Oudewater, Netherlands) to mitigate potential anaphylaxis due to microfilarial death.

A follow-up visit was scheduled two weeks after treatment initiation, but the owners visited the clinic a month later. Follow-up tests did not reveal any abnormalities (Table 1), except for slightly decreased hemoglobin in reticulocytes (21.8 pg). The blood smear test showed no presence of *Babesia* spp. protozoa or *A. phagocytophilum* morulae. The owners continued the treatment against dirofilariasis. Anaphylactic reactions were not observed during the treatment against *D. repens*, and prednisolone was discontinued due to increased polydipsia and polyuria.

## Discussion and conclusions

This case highlights the importance of comprehensive diagnostic evaluation, particularly in vector-borne diseases. Underdiagnosis may lead to the lack of therapeutic efficacy or death, and it poses an epizootic risk to other animals and humans. Co-infections often exacerbate clinical symptoms and negatively impact hematological and biochemical parameters [7]. Due to growing levels of health awareness in recent years, dog owners are increasingly likely to consult a veterinarian when behavioral abnormalities arise. For this reason, the observed changes may not yet be strongly expressed, as demonstrated by the absence of anemia in the presented case.

A similar case was reported in Warsaw, Poland, at a similar time (autumn of 2022) [9] and in Slovakia (report from 2014) [10]. In the first case, *D. repens* and *D. immitis* were confirmed by molecular analysis, Knott’s test, and rapid diagnostic assays. However, although *Anaplasma* spp. antibodies were detected in a rapid test, they were not confirmed by additional methods [9]. In the Slovakian study, 366 blood samples from clinically asymptomatic dogs with microfilariae were analyzed for co-infections. The samples were also analyzed for the presence of *Babesia canis canis* and *A. phagocytophilum* pathogens. One sample tested positive for all three pathogens [10]. This study focused solely on co-infections rather than the clinical presentation [10].

The diagnosis of granulocytic anaplasmosis is based on clinical symptoms, changes in hematological tests, positive PCR results, presence of morula in granulocytes, or a four-fold increase in antibody titer within four weeks [11]. Detection of *A. phagocytophilum* by PCR is possible two to three days post-infection, and antibodies appear after 10–22 days [12] or eight days after primary exposure and two to five days after morula formation [7]. A retrospective study conducted by Schäfer et al. [12] on 27,368 canine blood samples confirmed that antibodies are often detected in samples that test negative in the PCR assay, which points to contact with the pathogen. The positive PCR and negative serology most likely indicates an acute infection and a pre-seroconversion immune status. In turn, positive PCR and serology results may confirm the acute phase of anaplasmosis with seroconversion, or a new infection with antibodies from previous contact with the pathogen [12]. The detection of *A. phagocytophilum* by three different diagnostic methods most likely indicates an active infection with ongoing seroconversion or the presence of antibodies from previous exposure. It should be noted that antibodies can persist for several months [7], which may complicate proper diagnosis. The presence of morulae in granulocytes is indicative of an acute phase of infection, and the result should be confirmed by PCR to exclude misinterpretation of the blood smear and to enable differentiation from other pathogens, such as *Ehrlichia ewingii* [7].

Various species of ticks are considered to be the main vectors of anaplasmosis (*I. ricinus)* and babesiosis (*Dermacentor reticulatus)*. In the studied case, the owners did not report a tick infestation; therefore, potential exposure to one or more tick species could not be ascertained. Notably, *I. ricinus* has been reported as a potential vector not only of anaplasmosis but also of *B. canis* [13].

Molecular analysis of *I. ricinus* ticks from the North-Eastern Poland region revealed the presence of *A. phagocytophilum* in 5/423 ticks [14] and *Babesia* spp. in 8/104 ticks [15]. In the *D. reticulatus* species, *Babesia* spp. was detected in 7/37 [15] or 21/886 ticks [16], and *A. phagocytophilum* in 3/886 ticks [16]. In the mentioned studies, no co-infection with *Babesia* spp. and *A. phagocytophilum* was detected in ticks, therefore it can be assumed that the dog was exposed to more than one tick. Our previous studies also did not show that genetic material of *Babesia* spp. and *A. phagocytophilum* was detected in one tick or dog [2].

The blood smear analysis revealed the presence of microfilariae, and the rapid diagnostic test produced a positive result for *D. immitis* after 30 min. Heat treatment enhances the detection of *D. immitis* antigens [17]. However, a study by Sobotyk et al. [18] has shown that in co-endemic areas for *D. repens* and *D. immitis*, these tests have limited clinical value due to potential cross-reactions between pathogens.

Poland is already considered an endemic area for *D. repens*, which is confirmed by numerous reports [9, 19–21]. *D. repens* genetic material was demonstrated in 59/424 dogs from North-Eastern Europe [19]. There is no data on the prevalence of dirofilariosis in North-Eastern Poland.

In the present case study, doxycycline was administered not only to treat anaplasmosis, but also to eliminate the bacterial endosymbionts of *Dirofilaria* spp.- *Wolbachia* spp. There is evidence to suggest that the use of doxycycline to target *Wolbachia* spp. increases the effectiveness of the therapy [8]. When combined with macrocyclic lactones, doxycycline also inhibits microfilarial migration and reduces the risk of further transmission [22]. Treatment of anaplasmosis should last two to three weeks [7]. The use of corticosteroids in the treatment of anaplasmosis and babesiosis remains controversial. Current recommendations suggest that steroids should only be introduced in the absence of improvement or when symptoms of autoimmune disease are observed [7].

The therapeutic protocols for subcutaneous dirofilariosis rely mainly on macrocyclic lactones [8, 23]. The presence of *D. immitis* should be confirmed to determine the need for melarsomine therapy, according to the guidelines of the American Heartworm Society [24].

The PCR tests for anaplasmosis should be repeated several weeks after antibiotic therapy. Molecular analyses of *Babesia* spp. and *D. repens* should also be performed. In the analyzed case, the owners did not consent to additional tests due to the lack of any clinical and laboratory symptoms. This observation highlights a critical issue, namely the high cost of diagnostics and post-treatment monitoring. For some owners, clinical improvement alone is considered sufficient proof of therapeutic success. Unfortunately, this approach may contribute to the latent spread of infections in the local environment. This is an important aspect, because dirofilariosis and anaplasmosis have a zoonotic potential [25, 26]. It has been shown that dog owners may be seropositive for anaplasmosis [27, 28] and that a high level of seroprevalence of dogs for anaplasmosis suggests a higher risk of disease in humans [29]. Considering the safety of humans and animals, special emphasis should be placed on education of animal owners and control of treatment through molecular tests, which are characterized by greater sensitivity compared to microscopic tests. Studies should also be conducted to determine the prevalence of pathogens, especially those with zoonotic potential.

## Data Availability

No datasets were generated or analysed during the current study.
